# An Intervention Framework to Facilitate Psychological Trauma Management in High-Risk Occupations

**DOI:** 10.3389/fpsyg.2020.00530

**Published:** 2020-03-27

**Authors:** Bouwer E. Jonker, Lene Ilyna Graupner, Lizelle Rossouw

**Affiliations:** WorkWell Research Unit, Faculty of Economic and Management Sciences, School of Industrial Psychology and Human Resource Management, North-West University, Potchefstroom, South Africa

**Keywords:** trauma management programs, high-risk occupations, trauma management framework, intervention framework, psychological trauma, workplace trauma

## Abstract

Various psychological trauma management programs (PTMPs) are offered to assist employees who have been exposed to a traumatic event in the workplace. There is, however, limited literature available on how employees in high-risk occupations experience these programs. This study qualitatively explored the experiences of PTMPs from the perspective of employees working in three high-risk occupations. The purpose of this study was to explore the experiences of the participants in order to compile a framework that could help support and improve the productivity and wellbeing of employees affected by work-related trauma. The study used a qualitative research design based on an approach informed by interpretivism and social constructivism. A multiple-case study was used as research strategy to incorporate three sectors in South Africa, namely mining, policing, and emergency medical services. Data were gathered through semi-structured interviews and focus groups, and analyzed using thematic analysis. The findings across the three sectors showed effective strategies considered by participants to manage psychological trauma. These strategies include multiple counseling sessions, face-to-face counseling, regaining control, and receiving support. Strategies that were viewed as ineffective include inability of counselors to relate to the participants’ work environments, lack of involvement, lack of supervisor support, unavailability of counseling and specialized skills, premature resuming of duties, and a single-dimension approach. Based on the findings, an intervention framework is proposed to address psychological trauma in high-risk occupations.

## Introduction

There is major significance for organizations that implement effective psychological trauma management programs (PTMPs) to enhance employees’ work performance and psychosocial functioning (Terblanche and Van Wyk, 2014). High-risk occupational settings considered globally as some of the most dangerous are the mining, policing and emergency medical services (EMS), which often result in traumatic incidents (Adriaenssens et al., 2012; Kowalski-Trakofler and Vaught, 2012; Ménard and Arter, 2013). In South Africa, these work environments are experienced as particularly hazardous and traumatic (Wilson, 2011; Steyn et al., 2013). Employees are left with psychological trauma that hinders their productivity and threatens their well-being (Ward et al., 2006; Zungu, 2013). Psychological trauma can be defined as an experience of psychological wounding caused by an event outside the usual range of human experience (Mitchell and Everly, 2001). Such events include unexpected, often life-threatening, situations that may inhibit employees’ ability to respond in a normal way (Attridge and VandePol, 2010). Over recent years, various workplace-related incidents shook the world, such as terrorist attacks, civil wars, political violence, and state-supported oppression (Lemanski, 2004; Sorensen, 2012; Atvoli et al., 2013; North et al., 2013). These incidents warrant for employers to implement effective programs to manage workplace trauma.

## Literature Review

For purposes of this article, a PTMP is viewed as a collection of crisis intervention models, protocols, procedures, strategies, techniques, and practices that help manage psychological trauma in the workplace (Mitchell, 2004). According to Georganta and Montgomery (2019), workplace interventions entail single- or multi-component programs that implement strategies on individual, environmental, organizational, and group level (Le Fevre et al., 2006). An effective program to manage trauma in the workplace should cover the crisis continuum from pre- to post-crisis (Everly et al., 2000; Forbes et al., 2011). It is important that organizations act proactively, thereby ensuring employees’ wellbeing is promoted through effective interventions. Proactive programs to manage trauma in a workplace include a preparation phase for the organization and the employee (Mitchell, 2004). According to Forbes et al. (2011), a PTMP should be developed for a recipient organization in conjunction with the latter’s policies and procedures. The PTMP should then be promoted throughout the organization and appropriate training should be provided in trauma management at the various levels (Forbes et al., 2011).

During the crisis level frontline interventions may commence during or shortly after a traumatic event (Litz, 2008).

Literature suggests the following elements for an effective frontline intervention (Ruzec et al., 2007; Van Wyk and Edwards, 2005):

1. *Contact and engagement:* The helper makes contact with the affected person(s) and becomes engaged in the helping process.
2. *Safety and comfort (if applicable):* Address affected employees’ immediate concerns about safety. In this step, the various physical needs are provided for, such as medical care, food, or warm blankets.
3. *Stabilization:* The helper responds empathetically, in a safe and non-judgmental environment, to emotions the employee may experience.
4. *Information gathering:* Information on what happened is gathered in a sensitive manner.
5. *Practical assistance:* Practical solutions are provided to immediate challenges. Such assistance may include making contact with close family or friends and assisting with transport arrangements.
6. *Connection with social supports:* Employees who are vulnerable to isolation are identified. Non-judgmental social support from existing family and peer-group structures is encouraged.
7. *Information on coping support:* The affected employees are made aware of trauma-related symptoms that they may be experiencing, for example sleeplessness, nightmares, recurring thoughts, and difficulty with concentration. Such symptoms are normalized and the employees are provided with information that can help them cope after the traumatic event, such as avoiding being alone for long periods and talking to support networks about the trauma.
8. *Linking to support services:* A few days or up to 2 weeks after the event, follow-up takes place and affected individuals are reassessed. Employees who experience difficulty in the normal recovery process are offered trauma counseling or therapy.

Post-crisis or reactive interventions aimed at managing workplace trauma would traditionally include brief- and medium-term counseling, and long-term trauma therapy.

### Brief and Medium-Term Counseling

According to Kaminer and Eagle (2010), such counseling should only start a few weeks after the traumatic incident. Only employees who experience acute stress and post-traumatic stress symptoms should receive such interventions (Kaminer and Eagle, 2010). The following elements are recommended for an effective brief and medium-term trauma intervention (Eagle, 1998):

1. *Telling/retelling the story:* Requests the employee to recount the story of the traumatic incident in as much detail as possible. The clients therefore narrate facts, thoughts, feelings, and sensations. The aim is to help clients express unexpressed feelings and fantasies about the event and reduce the anxiety associated with it.
2. *Normalizing the symptoms:* Employees must relay the symptoms they experienced since the incident. Psycho-education on experienced trauma symptoms are provided and normalized. This is done to prevent the employee from forming irrational interpretations thereof.
3. *Addressing self-blame and survivor guilt:* These inner experiences are harmful to the employee’s self-esteem and should be dealt with in appropriate steps.
4. *Encouraging mastery:* The employees are encouraged to utilize available support systems. The helper should also teach employees techniques to reduce anxiety and structure time effectively.

### Long-Term Trauma Therapy

According to Kaminer and Eagle (2010), such interventions are associated with complex trauma involving post-traumatic stress disorder (PTSD). Long-term interventions can take months or years following a traumatic incident and may include the following elements to be effective (Kaminer and Eagle, 2010):

1. *Individual therapy:* Intensive intervention to address not only the effects of a single traumatic incident, but also contributing factors such as previous events and depression.
2. *Couples counseling:* Employees in relationships are encouraged to engage in such counseling. Traumatic stress can be strenuous for personal relationships.
3. *Group therapy:* As recommended by a therapist, employees may also benefit by attending sessions with other individuals who have experienced similar circumstances.

When frontline trauma interventions are implemented correctly, the levels of acute distress should stabilize and decrease. Furthermore, affected individuals should have been provided continuous care (Everly and Lating, 2017). Relatedly, employees’ immediate needs should be addressed (Schafer et al., 2016). According to McNally et al. (2003), and Rose et al. (2003), when frontline interventions are implemented incorrectly, they fail to prevent the onset of PTSD, and may even be harmful by re-traumatizing employees. After implementing brief and medium-term interventions, employees should reach a sense of closure, psychological stress should be alleviated, and a return to a level of pre-trauma functioning can be expected (Attridge and VandePol, 2010). In addition, anxiety levels should decrease, and the self-respect of employees restored (Eagle, 1998).

Since 2008, the International Society for Traumatic Stress Studies (ISTSS) withdrew its support for the use of psychological debriefing type of acute/frontline interventions (Litz, 2008; Foa, 2009). Critical incident stress debriefing is closely associated with psychological debriefing (Devilly and Cotton, 2003; Rose et al., 2003). In this regard, VandePol et al. (2006) point out that psychological first aid is favored by the World Health Organization [WHO] (2005) and the National Institute for Clinical Excellence (2005) above interventions that resemble debriefing. Psychological first aid is likewise recommended for use by the American Psychiatric Association (2004), the Australian Centre for Posttraumatic Mental Health (2007), and the Department of Veterans Affairs/Department of Defence (2004).

In contrast, the ISTSS recommends (Foa, 2009) that immediately after a traumatic incident, affected individuals should be offered practical support, information on possible stress reactions, and self-help guidelines. This should include information on accessing support from existing networks, as well as when and where to obtain further support if necessary. Frontline intervention should be based on existing and accurate assessment of needs. Furthermore, formal trauma intervention should not be mandatory for all employees exposed to trauma. The use of trauma support should be voluntary, except in instances where impairment is a threat to the safety of the individual or others. Foa (2009) adds that interventions should be developmentally appropriate, culturally sensitive, and contextually formulated by focusing on problems and coping strategies.

Therefore, managing trauma in high-risk occupational sectors with an effective intervention program should follow a holistic approach. The focus should be on offering a framework for understanding the complex and multi-dimensional concept of workplace health (Georganta and Montgomery, 2019).

In the present study, high-risk occupation sectors are viewed as jobs where employees are especially prone to exposure regarding traumatic experiences during the course of their work (Tehrani, 2004). These occupations, among others, include the mining, policing, and emergency medical sectors.

### Mining Sector

Comprises dangerous occupations due to the life-threatening situations to which employees are exposed (Bao et al., 2017). These hazards include underground earthquakes/tremors and rock-fall incidents, which may trap employees underground (Maiden and Terblanche, 2006; Stevens et al., 2006).

Zungu (2013) identified mining as a high-risk environment for trauma in South Africa, citing earth-fall and transport accidents as the cause of serious injuries and fatalities. The Department Mineral Resources’ 2017/2018 Annual Report recorded 88 mine fatalities in 2017. This figure signifies a 20.5% increase, year on year, as measured against the 73 mine-related deaths during 2016 (Department Mineral Resources: Republic of South Africa, 2018).

### Police Services

Members often are exposed to traumatic incidents and risk experiencing critical incidents in their work environment (Ellrich and Baier, 2017). According to Mostert and Rothmann (2006), policing is identified as one of the most dangerous occupations in the world. Steyn et al. (2013) report that hyper-arousal, as a result from exposure to traumatic events is the main predictor of suicide ideation in the South African Police Service (SAPS).

South Africa can be regarded as a country of persistent conflict that is characterized by various divisions (van Riet, 2020). According to the SAPS 2018/2019 crime statistics report, the total number (national) of reported contact crimes (crimes against the person, for example, murder, assault to cause grievous bodily harm (GBH), and assault are 617,210 (SAPS Annual Crime Statistics 2018/2019, 2020).

### Emergency Medical Services

These employees work in a high-risk environment (Roditi et al., 2019). EMS practitioners included in this study work as paramedics and EMS technicians who react to medical emergencies such as motor vehicle accidents and home responses; they are continuously exposed to witnessing severe injuries, death, suicide, and human suffering. As a result, they experience extreme and recurrent traumatic incidents that pose significant professional and emotional challenges (Adriaenssens et al., 2012). Relatedly, Bolm-Audorff et al. (2019) conclude that the risk of developing PTSD in EMS personnel is high.

Paramedics in South Africa are exposed to increased violent attacks, by criminal elements of the community (Mulaudzi, 2017). According to Evans (2018), nearly 30 cases were documented in the latter half of 2018 in the province of KwaZulu-Natal alone; similar incidents occurred in the Free State (Maphanga, 2019) and Gauteng (Chabalala, 2019). Monageng (2019) states that the high prevalence of attacks causes distress among EMS practitioners and disrupts service delivery to those who are most vulnerable. As a counter measure to this development, the South African Emergency Personnel Union (SAEPU) advised its 7,000 members to carry arms in preparation for the 2018 festive season (Evans, 2018).

The general objective of this article was to recommend an operational framework for psychological trauma management that may help improve trauma management in high-risk occupation sectors. This objective was attained by exploring the experiences involving PTMPs in high-risk occupations, namely the mining, policing, and medical emergency sectors, as viewed from the perspective of the employees.

## Materials and Methods

### Research Approach

An explorative, descriptive qualitative research design was used. The philosophical paradigms that guided the design of the present study and interpretation of the data are anchored in constructivism and interpretivism. An ontological assumption of constructivism and interpretivism is that multiple realities exist (Denzin and Lincoln, 2013). Nieuwenhuis (2016) explains that human experience can only be understood from within; reality is constructed during human interaction and meaning is created in the human mind; behavior is affected by knowledge of the social world; therefore, the social world and human knowledge are intertwined (Nieuwenhuis, 2016). The present study also employed phenomenology as a research approach. Employees were asked to describe their experiences of PTMPs that are offered in the mining, policing and EMS sectors in South Africa. This approach therefore engages the subjective experiences and interpretations of those exposed to the phenomenon (Trochim et al., 2016).

### Research Strategy

A multiple-case study strategy was used, which allowed the inquirers to explore employees within the mining, policing, and EMS sectors’ lived experiences of the PTMPs. The unit of analysis was the individual employees who participated in the study (representing the mining, policing, and EMS sectors) and who had first-hand experience with PTMPs.

### Participants

Three mining organizations selected from two provinces took part in this research. The mining companies were unrelated and chosen for their geographical positions. The research setting in the policing sector comprised three police stations from two provinces. The following crimes have a high incidence in these provinces: assault with intent to inflict GBH, murder, and sexual offences (South African Police Service [SAPS], 2020). Three police stations were selected where the murder and rape cases were the highest in the province, and also where the workforce was representative in terms of race, gender and age demographics. Two large EMS companies from one province and one medium-sized company from another province participated in the research. Purposive sampling was used to ensure employees from the mentioned three sectors, who were exposed to at least one traumatic incident in the line of duty, were included in the present study. Table 1 presents a summary of the participants.

**TABLE 1:** Characteristics of participants (*N* = 95).

Item	Category	Frequency		
		Mining sector *N*	Policing sector *N*	EMS sector *N*
Gender	Male	16	26	23
	Female	4	13	11
	MV	0	2	0
Ethnicity	African	14	30	13
	Colored	1	1	0
	White	5	10	21
Home language	Afrikaans	6	11	17
	English	1	0	7
	isiXhosa	3	4	0
	isiZulu	3	2	2
	Sepedi	0	2	2
	Sesotho	5	9	2
	Setswana	1	12	4
	siSwati	1	0	0
	Xitsonga	0	1	0
Years of work experience	1–10	12	8	29
	11–20	3	13	5
	21–30	3	14	0
	31–40	2	6	0

*MV, missing values.*

### Data Collection

In this study, a combination of semi-structured, face-to-face as well as focus group interviews was used as methods for data collection. The focus group interviews were conducted by the researcher and the interviews were conducted by the researchers and fieldworkers. The fieldworkers included three master’s students in industrial psychology in their final year of study. The fieldworkers assisted with the interviews in the EMS sector. An interview guide for the fieldworkers was developed with questions directed at participants’ experiences of the PTMP in their occupation sector.

Certain sectors in this study indicated that they could not avail the time for the participants for interviews since this was time consuming, and consequently the focus group method was chosen as data-gathering for those cases. At three police stations, the researcher was allowed to collect data from the shifts on duty. Therefore, conducting one focus group per police station was the best course of action to circumvent the issues of transport and poor service delivery. Forty police members participated in three focus groups conducted in the policing sector. At two mining operations, the work schedules of employees varied and scheduling individual interviews was the most practical procedure to follow; nine semi-structured interviews were conducted at the two mining operations. At one mining organization, the only way the researcher could collect data was to see one team as a collective before they went underground into the mine. A focus group was the most appropriate procedure to follow in this case. Eleven underground mineworkers participated in this focus group. The operational managers at two EMS branches allowed the researcher to collect data only if it did not curtail the branches’ ability to respond to emergencies. Individual interviews were therefore scheduled to ensure the availability of paramedics at all times. Thirty semi-structured interviews were conducted at these two EMS branches. Apart from the individual interviews, one focus group with four EMS members was scheduled to allow all participants an opportunity to respond.

Participants gave written consent for the electronic recording of semi-structured face-to-face interviews or focus group sessions.

### Data Analysis

The data in the present study were processed through thematic analysis. The researchers selected this method since it is compatible with the phenomenology paradigm and case study as research strategy (Botma et al., 2010). The six-step method of Creswell (2009) was applied to analyze the data of the present study: (1) the voice recordings of the semi-structured interviews and focus groups were transcribed verbatim into distinct Excel documents. (2) The researchers read through the data to obtain a general sense of the answers and an appropriate idea of how participants experienced the PTMPs in their respective workplaces as well as their recommendations to enhance psychological trauma management. (3) The researcher (BEJ) organized the data into sections of text before attaching meaning to the information. This process entails taking text data and dividing words, phrases, sentences, and paragraphs into categories. The supervisors helped identify the categories. (4) From the categories, the researcher and the supervisors created descriptions from the themes. Themes and subthemes were reported as principal findings of the study and conveyed multiple viewpoints of participants. The supervisors reviewed the themes and subthemes and therefore acted as appraisers of the findings. (5) The researcher reported the results of the study in a qualitative narrative. (6) The researchers interpreted the meaning of the data in accordance with findings from scientific literature. Based on the interpretation of the data, the main study leader developed a framework to address prevention interventions in high-risk occupations.

### Ethical Considerations

The researcher and the supervisors obtained ethical clearance from the research institution, and participating organizations granted the researcher permission to collect data. Due to the sensitive nature of the study, participation was voluntary and participants could withdraw from the study at any time, without repercussions. Furthermore, all participants were ensured anonymity to guarantee their privacy. As a precautionary measure, trained psychological trauma counselors were placed on stand-by in the event participants became uncomfortable during the data collection phase. All participants were debriefed after data collection to ensure no contributor was left vulnerable or has experienced unresolved issues.

## Findings

This section outlines the collective results derived from the data analysis conducted for all three working environments. The results revealed two categories, from which themes and subthemes were extracted and corroborated with direct quotations from participants from all three sectors. Responses in African languages (mining sector) were translated into English by an accredited language practitioner. Frequencies (i.e., numbers) were not assigned to the extracted themes and sub-themes. In qualitative research the objective is not to quantify results as is done in quantitative studies, however, to rather report unique experiences of participants. Both the constructivism and interpretivism paradigm, as employed in this study is in support of this viewpoint. The objective of these two paradigms is to understand the unique and multiple realities and experiences of participants. Therefore, participants can be exposed to the same phenomenon (i.e., PTMP’s), however, their experiences thereof may differ. The researchers therefore decided to rather report all the unique and multiple experiences of the participants.

### Category 1: Experiences Regarding Psychological Trauma Management Programs

Results of this category were obtained by asking the participants about their experiences of the PTMPs in their respective workplaces. This question allowed a deeper understanding of how employees per sector experience the programs. In Category 1, the two main contrasting themes were identified as *effectiveness* of the PTMPs and *ineffectiveness* of these programs and are discussed next. Refer to Supplementary Tables A1, A2 for a more extensive overview of the responses of the participants for each theme.

#### Theme 1: Effective PTMPs

**FIGURE 1:**
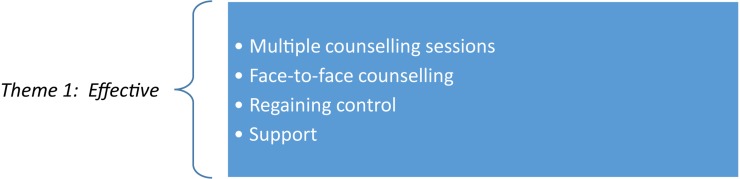
Psychological trauma management programs viewed as effective.

An interesting finding was that no participants from the policing sector could indicate positively how they experience the PTMP within their organization. Overall, it can be concluded that the participants in all three sectors generally had more negative than positive experiences of PTMTs. However, particularly in the policing sector, no participant felt that the management programs helped them manage trauma effectively. Figure 1 provides the common subthemes that emerged from the first theme in Category 1 for the two sectors involved:

From Figure 1, it can be seen that in both the mining and EMS sectors, the participants cited multiple counseling sessions and referrals to a psychologist as *effective* means to manage traumatic experiences – as illustrated by the excerpts below.

“I had four or five sessions with them, but they didn’t talk, no other funny things. Every time I walked away from there, it felt more and more that a weight is lifted off my shoulders.” (Participant 9: Male. 36 years old. Mining sector)

The participants spoke highly of the PTMP’s effectiveness, particularly when it took the form of face-to-face counseling. One participant mentioned that the counselor helped him verbalize his traumatic experience, as the following excerpt points out:

*“In my initial contact with them, I went to five sessions with a clinical psychologist and we talked about the incident and she taught me coping mechanisms … I still remember and I still use them.”* (Participant 28: Female. 32 years old. EMS sector)

Regaining control was an important benefit the paramedics described after utilizing the PTMP. One group member stated that he struggled previously to control his temper. After participating in the program, he found that he did not lose his temper that often anymore. Another paramedic explained that he did not suffer sleepless nights anymore, which often resulted from traumatic experiences at work.

The participants from the above-mentioned two sectors indicated further that they found support as efficient to manage their traumatic experiences. One participant explained that the support he received from the social worker had a harmonizing effect on them, after they were exposed to a traumatic incident. He indicated that the practitioner went out of her way to support them. Another participant attested that the social worker helped them understand and cope with the traumatic incident where one of their colleagues died in a mining-related accident. One respondent clarified that the social worker helped the crew become “normal” again.

Participants from the EMS sector indicated that they value colleague support as a way to manage psychological trauma. Turning to a colleague for support is known as the “buddy system” in the EMS sector. One participant explained that only paramedics can truly understand the nature of EMS work. Therefore, it is better to ask the opinion of someone who has had a similar experience. Another participant reported that she talks to more experienced colleagues after a difficult scene and this process makes it easier for her to cope, as seen in the response below:

*“They have got more experience. I just talk to them. And then the more you talk, the easier it gets. The more it fades away … You can’t even talk about it at home, because they don’t understand what is happening or what happened.”* (Participant 33: Female. 35 years old. EMS sector)

#### Theme 2: Ineffective PTMPs

**FIGURE 2:**
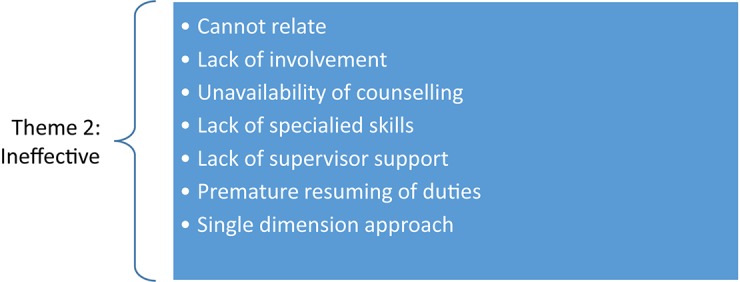
Psychological trauma management programs viewed as ineffective.

Several subthemes were captured from all three sectors in the second theme in the category: experiences of PTMPs as *ineffective*. The subthemes are shown in Figure 2.

Figure 2 shows that a common subtheme for all three sectors was that participants felt the counselors were unable to relate to them, which caused the trauma management program to be *ineffective*. In the mining sector, one participant indicated that the counselor was not on site, and therefore could not relate to the mining environment. This participant experienced the counseling process as impersonal and felt he could not trust the counselor. In the policing sector, especially, the participants felt strongly that the counselors do not understand their experiences, and therefore are unable to provide an effective service, as the following response shows:

*“The caseworker … has never had to pick up a body part on an accident scene. He never had to pick up a child that has been cut in half and place him on a trolley. He has never been shot at in his life. He has never smelled how it smells when a, when a human being is burning, and they want to come and debrief me. You cannot debrief me if you do not know what is going on outside.”* (Participant 23: Male. 42 years old. Policing sector)

Similarly, in the EMS sector, the participants indicated they felt the counselors could not relate to paramedics’ experiences. One participant pointed out that counselors do not understand what EMS does, as is evident from the excerpt below:

*“I don’t think the guys from the counseling company understand what we are doing, they sit behind a desk …”* (Participant 20: Male. 33 years old. EMS sector)

Responses from participants in the EMS sector showed that they preferred to talk to one another after a particularly distressing incident. The participants indicated that they would rather talk to friends and family instead of making use of the PTMP. One participant mentioned that talking to friends about a bad incident they had experienced made them feel better. They also felt more appreciated and validated for the work they do – almost like being a hero. Another group member stated that after a traumatic incident, he confides in family members, namely a wife who is a trained counselor. A different participant talks to her father who is also working in EMS.

In contrast, significantly, participants in the policing environment felt that counselors not being independent to the organization made the program ineffective. The participants indicated that counselors’ objectivity is compromised since they work for the same organization, as the following excerpts clarify:

*“You have people (counselors) that’s working for the police; they are looking after the police problems, your problems start to be the second, or not prioritized.”* (Participant 35: Male. 32 years old. Police sector).

Another common theme across the three sectors was that counseling services are available on request and when needed most. However, it is clear that the participants needed involvement from counselors as opposed to being off site and only available telephonically. In the mining sector, they indicated that mere telephonic counseling after an incident was ineffective. One participant explained that the process is impersonal since the counselor is not available on-site. Another participant reported that after an incident, she had to phone the counselor herself only to be told she needs to see a psychologist, who often was unavailable when she made contact by phone.

Similarly, in the policing sector, the participants indicated that they regarded the PTMPs as ineffective since no counseling was offered after a traumatic event. One participant related that she (as detective then) was denied access to counseling after a colleague (close friend) was shot because these interventions were offered only to uniformed police members. Other participants attested that the counselors were not involved at all; they have only heard about counseling after trauma, but were not offered it. In the EMS sector as well, a number of paramedics indicated the absence of PTMPs at their workplace – no counselor nor an emergency helpline to contact should they require counseling.

A further subtheme emerged from this category, especially in the policing and EMS sector. Participants revealed that counselors often lack proper skills, thereby rendering the trauma management program ineffective. In the policing sector, the participants indicated that counselors have insufficient knowledge and skills regarding psychological trauma management. One participant observed that the counselors refer police members to doctors and psychiatrists for medication without attempting to find the root of the problem themselves. In the EMS sector, the participants point out that counselors are not trained properly to provide clients a better perspective, as is illustrated by the following excerpt:

*“Like the people that one talks to are not really trained to give you a better perspective.”* (Participant 11: Male. 25 years old. EMS sector)

In the mining sector, a specific subtheme that emerged was that participants viewed the PTMPs as ineffective since they lack support from their supervisors. Mineworkers indicated that the lack of physical presence (support) could be described as “a disgrace, poor and sad.” According to participants, the way supervisors approach them, or talk to them after a traumatic incident, shows a lack of sensitivity and understanding about psychological trauma. One respondent mentioned that their supervisor did not even look in on them the day the accident occurred, he only came the following day. The excerpt below demonstrates the need of the respondents to have support from their supervisors:

*“I used the word poor because sometimes, if we are working together and someone got sick. He’s lying in hospital. The supervisors or anyone, they don’t come and visit you or any employee of the company comes to see how you are doing.”* (Participant 17: Male. 37 years old. Mining sector)

Another subtheme captured across the three sectors indicated that the participants felt they are required to return to work prematurely to resume their duties after a traumatic incident. Underground mineworkers regarded the PTMPs as ineffective, because supervisors were allowed to send traumatized employees back to the same worksite where the incident took place, too soon after the incident. The response below illustrates participants’ concern of being well enough to return to work:

*“After the event, the way the counseling was done it was too quick. It wasn’t done the way it’s supposed to be done; and then you cannot say, ‘Today there is a seismic event there was a fatal there, ‘and the supervisor says, ‘Go there tomorrow and fix one, two, and three.’ You still think that person is in the right state of mind to perform any job underground?”* (Participant 20: Male. 37 years old. Mining sector)

In the policing sector, the participants were critical of a singular approach to address traumatic experiences through formal debriefing offered to members after a traumatic incident. They found such debriefing insufficient to help them recover from psychological trauma, as illustrated by the excerpt below:

*“If an incident happens a group, a person is called for debriefing, they ask you questions and look into your eyes and then you go home. And that’s the end of the story. That’s where your trauma counseling in the police stops.”* (Participant 13: Male. 51 years old. Police sector)

### Category 2: Recommendations to Improve the Effectiveness of PTMPs

The second category that emerged from the data showed that the participants from the mining, policing, and EMS sectors indicated their preferred type of PTMP, which they consider effective interventions to manage their trauma experiences. Table 2 presents the themes and subthemes, with verbatim responses on collective interventions the participants suggested across the three sectors.

**TABLE 2:** Collective needs regarding PTMPs across the mining, policing, and EMS sectors.

		Responses		
Theme	Subtheme	Mining sector	Policing sector	EMS sector
Prevention/pre-crisis	Proactive	*“Psychologists must come and inform us and give us more information on what we can do and what process we can follow when we go through a traumatic experience. We need to know who we can consult. Errors are caused by a lack of information.”* (Participant 15. Male. 39 years old)	*“They should maybe just visit us, maybe twice a week, because now we don’t see them at all. Maybe twice a week or once a week then they must just visit our parades and everything and ask if everything is still well and then if we need them then we should come to them.”* (Participant 4. Male. 31 years old)	*“A program before on hand that prepare you for this and this and that. I just wish there could be some emotional training on that. The first time I saw a person died in my face I have never seen that.”* (Participant 33. Female. 35 years old)
	Create awareness	*“There should definitely be more awareness about it. A person knows about the program, but someone does not always know what it is about, or in what way it can help you … They must maybe put up poster or such thing to create awareness. I don’t even know what the program’s name is.”* (Participant 7. Male. 48 years)	*“But most of us are not aware. We are not aware about this. You see on those psychological factors we are not aware of that.”* (Participant 7. Female. 26 years)	*“It is very important for one to just become knowledgeable and then make a thorough research to know what this is really.”* (Participant 8. Male. 41 years old)
	Physical activity/hobby	–	–	*“If I had a bad day at work, I get my load off by doing mixed martial arts.”* (Participant 2. Male. 27 years old)
				*“I always say you need to have a hobby. I have parrots that keep me busy and I also build up motorbikes at home and those types of things.”* (Participant 9. Male. 43 years old)
Recuperative/acute	Regular psychological support	*“The mine must get a psychologist who is more approachable, or who has more time to see people at the mine … you can arrange an appointment with him/her. Then you know you see the psychologist regularly.”* (Participant 4. Female. 36 years old)	*“Maybe twice a week or once a week then they must just visit our parades and ask if everything is still well and then if we need them then we should come to them.”* (Participant 4. Male. 31 years old)	*“I think it needs to be put in place. It is not normal what we see, so I think they need to get a trauma counselor.”* (Participant 4. Male. 32 years old)
	Independent psychological service provider		*“When you have people that’s working for the police they are looking after the police problems, your problems start to be the second, or not prioritized.”* (Participant 35. Male. 32 years old)	–
	Psychological support on site	*“We need someone that can talk face-to-face with us. That can see our pain, not over the phone.”* (Participant 8. Male. 25 years old)	*“I think they must be available at station level or accounting station level on a 24-h basis, not standby, they must be on a shift 24 h a day.”* (Participant 1. Male. 51 years old)	*“We used to have that thing called debriefing after everything that happened we sit at the table and then we will discuss whatever happened, so that was therapeutic for everyone there; it is accessible at any time.”* (Participant 3. Male. 39 years)
	Medical support/evaluation	*“It takes long, because it goes from the Safety to the manager. Then to the HR Department. So it has to go through all the necessary steps before you can qualify for psychological treatment. I do think that the longer it takes, the deeper the trauma becomes and the more difficult it will become for someone to talk about the trauma.”* (Participant 10. Male. 29 years old)	*“My view is strengthen the psychological leg of the medical aid and make it compulsory that every member once a year must see a psychologist and psychiatrist for an evaluation to make sure inside the service.”* (Participant 13. Male. 51 years old)	–
Post-crisis	Management support	*“We need our supervisors to be close to us, not too close to us if it is something for the production only, even if the event of the death of someone, the event of bad things we need them to come close to us, all the time.”* (Participant 19. Male. 34 years old)	*“Our commanders they read our dockets every day, they know what we did, what we dealt with, I think from them they can make an appointment with EHW A police manager’s job is and part of the responsibility if you are a manager, is to look after your members or the people that are serving and to make sure that they can operate effective.”* (Participant 1. Male. 51 years old)	–
	Peer support/family support	–	–	*“I can add like if you are the crews, if we see someone is not coping, we can talk. discuss things and give her or him some advice how to deal with it.”* (Participant 17. Female. 28 years old)
				*“And sometimes I talk with friends and family, ‘ Listen here, today I saw one two three.’ Then it becomes less of a bad thing because I have already spoken about it.”* (Participant 8. Male. 41 years old)
	Continuous development and training	*“Psychologists can do a workshop with case studies to inform the employees how the process works and that the process really helps.”* (Participant 15. Male. 39 years old)	*“I think they need to be empowered. They don’t develop themselves, so they need to be empowered, more experienced.”* (Participant 34. Male. 58 years old)	*“A program before on hand that prepare you for this and this and that. I just wish there could be some emotional training on that. The first time I saw a person died in my face I have never seen that.”* (Participant 33. Female. 35 years old)

From Table 2, it is clear that the three sectors share common themes on the PTMP they would prefer in their occupations. The data for the first theme centered on a *preventative* approach. This involves actions dealing proactively with psychological trauma before an incident occurs. Examples are: creating awareness through marketing material and sharing information to gain knowledge on psychological factors relating to trauma. The paramedics preferred physical activity and having a hobby as a way to deal with psychological trauma.

The respondents indicated various PTMP interventions they would prefer on a *recuperative* level, during the acute phase of the crisis. Participants across all three sectors indicated that they would prefer more regular psychological support as well as services available face to face and on site. One sector (policing) indicated that they would prefer an independent service provider. Medical support and evaluations were further mentioned as forms of preferred intervention. Specific reference was made to a medical aid division that provides psychological care.

On a post-crisis level, the participants in the mining and policing sectors indicated that they value management support, while the participants from the EMS sector highlighted the value of peer and family support. Further suggestions were continuous development and training where employees could be empowered and informed.

## Discussion

The present study focused on the experience of employees in the three sectors of high-risk occupations regarding PTMPs. The findings indicated two categories in this regard, which are expounded below.

### Category 1

The first category relates to participants’ experience of the PTMPs in their various sectors. Two main themes were captured from this category: PTMPs viewed as *effective* and PTMPs viewed as *ineffective* in managing psychological trauma.

Across the three sectors, findings showed that the respondents considered multiple counseling sessions as effective to help manage psychological trauma. In their review and meta-analysis, Roberts et al. (2009) indicate that multiple counseling sessions, especially cognitive-behavioral therapy, seem more effective than supportive counseling. The respondents from all three sectors indicated that they prefer regular, face-to-face and multiple counseling sessions to manage psychological trauma. This view corresponds with findings from Boshoff and Strydom (2017), who indicate that counseling services in the police sector consist mostly of a single-session intervention that lacks long-term therapeutic effectiveness. Mining companies in South Africa generally do not employ clinical psychologists on a full-time basis. Certain mining organizations contract clinical psychologists as part of an external network of service providers (Ntimbane, 2018, Personal communication). Traumatized mineworkers are referred to psychologists through doctors, wellness clinics or the human resource department of the mining company. A number of mining organization in South Africa, however, do employ social workers who render support and counseling services (Employee Assistance Program counselors) to mine employees (Maiden and Terblanche, 2006).

From the results, it is evident that the participants valued face-to-face counseling more than the telephonic mode. According to Stickley (2011), non-verbal communication is important to create a therapeutic space. Observing non-verbal communication is, however, impossible when counseling is done telephonically. The participants viewed support from social workers (mining sector), as well as colleague support (EMS sector) as a valuable part of a PTMP. According to Inbar and Ganor (2003), employees in the helping professions utilize self-made support systems from social networks and other professional colleagues. In the EMS sector, the buddy system is a peer-support structure where colleagues are encouraged to share their concerns and experiences mutually (Mishra et al., 2010). Alexander and Klein (2001) as well as Jonsson and Segesten (2004) point out that implementing the buddy system in the EMS sector has been effective by molding organizational culture, thereby preventing or mitigating the onset of burnout.

Paramedics found the PTMPs to be effective, since it helped them regain control after experiencing the negative effects of a traumatic event. Sanderson (2013) explains that EMS personnel use a degree of dissociation (screening out the environment) for improved functioning when they encounter risk, danger or chaos. This can be a highly adaptive strategy to strengthen concentration and contain emotions. A high degree of dissociation (i.e., clinically) can, however, cause a person to lose contact with reality, the body, or sense of self for a period of time. Clinical dissociation is often associated with a feeling that individuals have lost control over actions, feelings, memories, and thoughts (Sanderson, 2013). From the findings, it seems as if trauma counseling helps some paramedics regain a sense of control.

A significant and alarming finding from the policing sector was the lack of positive feedback on participants’ experience of the PTMPs in the SAPS. Generally, the participants across the sectors indicated more negative than positive experiences of these programs. However, in the policing sector specifically, no participant felt the PTMPs help them manage trauma effectively. Cooper and Cartwright (1997) point out in their article that organizations should recognize counseling involves expert skills and requires extensive training. Particularly participants in the policing sector showed a reluctance to trust the counseling services. These participants found that the counselors do not always maintain confidentiality. The findings indicated further that the counselors cannot relate to police work, as they were not exposed to the same traumatic incidents such as life threatening situations and disturbing scenes. Macqueen (2019) states that principles such as confidentiality, integrity, and honesty require revision. The reason is that practitioners more often work in favor of the providers of psychotherapy than the clients and patients. Privacy and confidentiality are essential for counseling to be effective (Bond and Mitchels, 2014).

According to participants, a shared operational experience would be having a better understanding of a police official’s work environment. It is therefore understandable why certain participants argued that psychologists with actual policing experience would be more effective as trauma counselors. However, exposing counselors to life-threatening situations and disturbing scenes may traumatize those who are supposed to render trauma services to their clients.

A major concern for the participants in both the mining and policing sector was supervisor support. Jansen and Brent (2005) explain that mineworkers are more likely to move beyond their job descriptions if they are satisfied with their jobs, perceive their supervisors to be supportive and understanding, and consider that they are treated fairly by the organization. Supervisors will therefore benefit from supporting employees during and after traumatic events. Employees will likely respond positively to such support. According to Stephens and Long (2007), in a sample of police officers (*n* = 517), supervisor support correlated strongly to lower PTSD symptoms. Bakker et al. (2003) found that employees who experience social support in the workplace seem more dedicated and committed to their organization.

From the results, it is clear that the participants valued a holistic multi-dimensional approach to psychological trauma management. However, police members from the present study perceived debriefing as insufficient. According to Everly et al. (2000) and Mitchell (2004), follow-up and referral are important aspects of critical incident stress management (CISD). Boshoff and Strydom (2017) point out that CISD in the SAPS consists mostly of a single-session intervention that is not therapeutically effective in the long term. Employees reported that they were referred too easily to doctors and psychiatrists for medication instead of being offered counseling to identify the root of the problem and addressing it accordingly.

The present study highlighted the importance of proper training and skills development for helping professionals. Participants pointed out that counselors lack proper skills, which cause PTMPs to be ineffective. In the policing sector, the participants indicated that the counselors had insufficient knowledge and skills to handle psychological trauma management, while interviewees from the EMS sector indicated that the practitioners they talked to were not properly trained to help improve their perspective on recovery from psychological trauma. This finding is in accordance with a recommendation made by the Society for Industrial and Organizational Psychology (2019), that employee counseling and individual rehabilitation are potential important process capabilities and skills that preferably need to be included in the training of industrial psychology practitioners. According to Forbes et al. (2011), employees at the various levels in an organization (including supervisors) should be educated about psychological trauma and their role in times of crisis. It could be advisable to train peer educators, especially in the policing sector, since the peers can relate to each other, a characteristic most participants indicated to be important in a PTMP. The paramedics also indicated that training is linked to improved management of their psychological trauma. This view can be explained by the volatile nature of emergencies where paramedics’ actions and decisions make the difference between life and death. Findings from a sample of disaster workers showed that practical, procedural training in the job beforehand and group support during it, is more effective than psycho-education on stress management (Alexander, 2005).

Furthermore, participants highlighted adequate recovery time as crucial. They felt they have to resume their duties prematurely and do not have sufficient recovery time from psychological trauma. According to Cooper and Cartwright (1997), organizations should focus on workload and work pace. The organization should take care to allow recovery time from demanding tasks by allowing employees more control over the pace of work.

### Category 2

The second category, emerging from the results, points to participants’ needs about elements that should be included in a PTMP. From the results, it is clear that an effective PTMP should include three intervention levels, which could be compiled into a generic framework, but should also include personalized approaches. Table 3 indicates that participants from all three sectors recommended preventative interventions, also specific interventions during the crisis and recuperative interventions. The three levels that emerged from the participants can be illustrated as follows:

#### Pre-crisis

On preventative level, participants suggested certain proactive interventions before the crisis takes place. According to Everly et al. (2000) and Forbes et al. (2011), an effective workplace PTMP should include elements ranging from pre- to post-crisis interventions. Pre-crisis entails a preparation phase for both employees and the organization (Mitchell, 2004). This preparation includes elements such as developing a program aligned to the organization’s policies and procedures; marketing the program; and training at the various organizational levels (Forbes et al., 2011). These elements were also found in the current study; the participants indicated that knowledge about trauma and emotional reactions would help them deal with psychological trauma more effectively. This view is in line with Everly et al. (2000), Forbes et al. (2011), and Mitchell (2004), who advocate pre-crisis preparation that includes psychological trauma education and training at the various levels in the organization.

The participants in the EMS sector specifically reflected on physical activity and hobbies that help them manage psychological trauma. Salmon (2001) argues that physical exercise reduces anxiety and depression and offers resistance to the physiological and psychological costs of stressors.

#### Acute

Regarding a recuperative level, such interventions should address the employees’ needs during the crisis. The participants indicated that regular psychological support (across the three sectors) is necessary during the crisis phase. Only in the policing sector did participants indicate that they preferred an independent psychological service provider. Kaminer and Eagle (2010) point out that *post-crisis* interventions should include frontline as well as brief- and medium-term interventions, but also long-term trauma therapy. Roberts et al. (2009) report that multiple trauma counseling sessions were effective to address the negative impact of traumatic incidents on individuals. Mineworkers also recommended that the process of referring traumatized employees to psychologists should be accelerated during this phase. Badenhorst and Van Schalkwyk (1992) recommend that, within a South African context, trauma management should be implemented as soon as possible after a mining-related accident.

Furthermore, participants in the EMS sector did not require any medical support or evaluation themselves. This is interesting, since they occupy a profession in the medical environment, as opposed to the other two sectors, policing and mining, who viewed medical support and evaluation as an important intervention to recover from psychological trauma. Mabunda and Idemudia (2012) recommend that the mental health of workers in South Africa should be evaluated regularly to detect, among others, signs of anxiety, stress, and depression. EMS employees suggested that continuous training should be provided to them since it is linked to the management of their psychological trauma during the acute phase. This view can be explained by the volatile nature of emergencies where paramedics’ actions and decisions may imply life or death. Practical, procedural training in the job beforehand and group support during work were found to be more effective for disaster workers than psycho-education on stress management was (Alexander, 2005).

#### Post-crisis

On the third level, after a crisis took place, the organization’s support clearly enhances employees’ wellbeing when recovering from psychological trauma. Management support was found to be especially important for the mining and policing sectors. In contrast, participants from the EMS sector did not highlight supervisory support as a means to manage psychological trauma. However, the paramedics considered the valuable support those they received from their co-workers, friends, and family. Interestingly, the mining and policing sectors made no mention of co-worker or family support. Angelo and Chambel (2013) indicate that supervisory training is important in techniques to help strengthen peer support in the workplace. According to Gilbreath and Benson (2004), supervisors who are perceived to employ positive behaviors, and rarely negative conduct, contribute to the psychological health of subordinates. Furthermore, Inbar and Ganor (2003) point out that people in the helping professions make use of self-made support systems from social networks and other professional colleagues. This is also evident from the present study where EMS employees use the “buddy system.”

**FIGURE 3:**
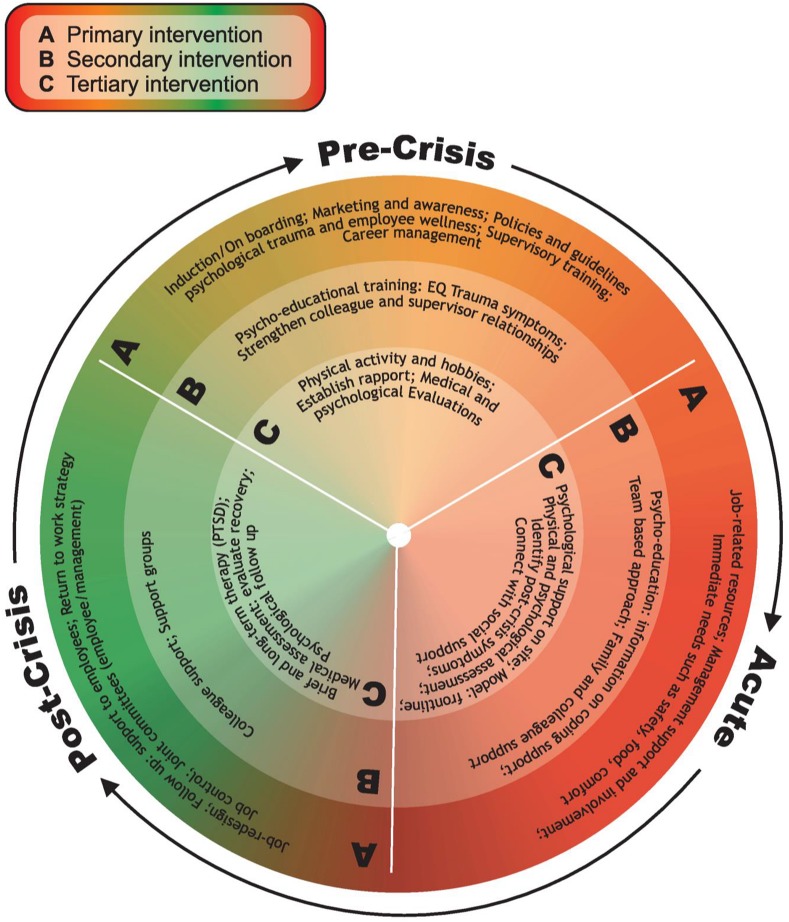
A conceptual intervention framework to address psychological trauma in high-risk occupations.

From the aforementioned discussion, based on the findings of this study, a framework is conceptualized and presented in Figure 3. The aim is to address psychological trauma management in the workplace utilizing this framework on the three levels identified by Hurrell and Murphy (1996), namely primary, secondary, and tertiary prevention interventions. The conceptual framework, deliberated next, links the pre-crisis, acute, and post-crisis phases to the three levels of prevention interventions for high-risk occupations.

Hurrell and Murphy (1996) refer to the use of “primary prevention interventions to reduce risk factors or to change the nature of the job stressors” (p. 339). In the current study, primary prevention strategies that link to the participants’ responses include a preparation phase such as induction to the organization, marketing and creating awareness of trauma-related symptoms and interventions, putting policies and procedures in place to address psychological trauma and employee wellness, supervisory training and a career management process for employees. During the acute phase of a crisis, the primary level interventions include having sufficient job-related resources, the availability of management support and addressing immediate needs, such as providing safety, food, and comfort. The post-crisis phase allowing job-redesign was a focus point in the study, also following up on employees who were involved in the incidents and providing continued support. An important aspect on primary intervention level is to have a return-to-work strategy in place to guide commencing with duties at a suitable stage. The participants indicated that having job control assisted them during the post-crisis phase and that a joint decision-making process with management was important.

*Secondary* interventions deal with the consequences rather than the causes of stress, which may be innate to the organization’s culture, and focus on increasing awareness and improving employees’ skills in stress management (Cooper and Cartwright, 1997). Strategies to improve stress management include activities that empower employees to identify symptoms of stress, thereby adding to the employee’s physical and psychological resources. The intervention level of secondary prevention, however, assumes that the inherent nature of the organization may not change; for example, the nature of police work is likely to stay the same. Therefore, the inherent nature of the work will continue to be stressful and therefore employees have to increase their tolerance and resistance to stress. In this study, for the pre-crisis phase, the participants referred to psycho-educational training in topics such as EQ and trauma symptoms to empower them. Furthermore, having colleague and supervisor support was especially significant to the participants during this phase. During the acute phase of a crisis, having knowledge on coping support and following a team-based approach to cope with the crisis were effective psychological trauma management strategies. The participants also found family and colleague support especially helpful to cope during this phase. Colleague support was also effective in the post-crisis phase; however, support groups were mentioned to be of value to assist the employees with their recovery.

*Tertiary* prevention interventions are concerned mainly with the treatment, rehabilitation, and recovery of those individuals who have undergone or currently are suffering from serious ill health due to stressful events (Cooper and Cartwright, 1997). Typical interventions focus on counseling services, and procedures to facilitate and assist employees’ recovery and return to work strategy (Cooper and Cartwright, 1997). In this study, during the pre-crisis phase, the participants indicated that it helped them to be physically active or to have a hobby to focus their attention on; having established rapport with significant others helped them during this phase as well as going for medical and psychological evaluations as a means to act proactively. During the acute phase of a crisis, having psychological support on site was especially helpful, also utilizing the frontline intervention model during counseling, identifying trauma symptoms and connecting employees with their social support were helpful during this phase. After the crisis took place, the tertiary interventions the participants found helpful to address ill health focused on brief and long-term therapy to address PTSD symptoms, going for medical assessments to evaluate their recovery, and having psychological follow-up sessions.

The framework is outlined in Figure 3.

### Limitations of the Study

Firstly, the participants in the present study were mostly male. The mining, policing and EMS environments in South Africa are, however, male-dominated, which imply that females are under-represented. One major challenge the authors were faced with was obtaining participation and approval from the sectors where females were mostly more representative, typically the office-based employee assistance support services. Unfortunately, these sectors declined participation. Despite the gender imbalance, this study still provides useful insights that can be applied more broadly in the field of psychological trauma management. Secondly, data were collected from only two of the nine provinces in South Africa. More provinces could have been included. This was done, however, since the study was exploratory in nature and sufficient cases were accessible to the researchers in the two provinces to investigate the phenomenon. Lastly, other sectors such as the fire-and-rescue services and the cash transportation industry could also have been included in the present study. These settings can also be regarded as high-risk occupational environments and should be eligible to trauma support services.

### Implications

This study contributed to organizations in the mining, policing and EMS sectors by providing a contextualized understanding of the types of events their employees regard as traumatizing and how these events should be addressed and managed. For the mining sector, management should take note that management support was mainly a focus for employees when recovering from psychological trauma. Furthermore, it became clear that for participants, the management’s involvement, especially with funerals and visits to staff members in hospitals after traumatic incidents, was deemed as having a positive effect. Implementing such requested support could make a significant difference to the way the employees experience workplaces. In the policing sector, the main focus was more on obtaining an independent confidential psychological service. Management in the policing sector could consider such a service and also address the stigmatization about receiving counseling.

Decision-making parties in the EMS sector should consider expanding on the perceived support from families and co-workers. The implication may be that signs and symptoms of psychological conditions such as acute stress disorder, PTSD and depression may not be recognized in time. Such support systems are helpful, but may not be trained adequately in diagnosing and treating the aforementioned conditions. An external service provider who can help a paramedic regain control of feelings, thoughts and behavior, would translate into an effective PTMP in this case.

Future studies should include other high-risk occupations such as fire and rescue services, also including more females, Indian and colored participants in its sampling. Another future research perspective could be to explore the employers’ point of view. A practical implication and strength of the study is evidenced in the creation of a contextualized understanding of the participants’ experiences regarding the current PTMPs and the recommendations to improve the effectiveness of such interventions. This leads the way toward a conceptual framework proposed for the management of PT in high-risk occupations in South Africa. Finally, a future research initiative could include to verify how the *ad hoc* framework could possibly support and improve productivity and wellbeing of employees affected by work-related trauma. In this way, they can establish a positive intervention framework for high risk occupations.

## Conclusion

Organizations can benefit largely from implementing relevant PTMPs. Employees in high-risk occupations are often exposed to life-threatening and dangerous incidents, which may lead to psychological trauma. Managing these traumatic experiences should be facilitated by a program that incorporates multiple components ranging from pre- to post-crisis.

## Data Availability Statement

The datasets generated for this study are available on request to the corresponding author.

## Ethics Statement

The studies involving human participants were reviewed and approved by the Ethics Committee: Economic and Management Sciences. The patients/participants provided their written informed consent to participate in this study.

## Author Contributions

All authors are registered industrial psychologists who share an interest in intervention research and qualitative research. BJ is a Ph.D. student who conducted this study as part of his doctoral thesis, responsible for the data collection, data interpretation, and writing of the thesis. LG is a professor and main supervisor of the Ph.D. study, helped to conceptualize the study, a part of the data collection and the writing up of the manuscript for publication purposes. LR is the co-supervisor and assisted with the conceptualizing of the study and data interpretation.

## Conflict of Interest

The authors declare that the research was conducted in the absence of any commercial or financial relationships that could be construed as a potential conflict of interest.
