# Supplementary material for: An Intervention Framework to Facilitate Psychological Trauma Management in High-Risk Occupations
Source: Front Psychol. 2020 Mar 27;11:530. doi: 10.3389/fpsyg.2020.00530 (PMC7118223; doi:10.3389/fpsyg.2020.00530)
Supplement: Supplementary file 1 [file Data_Sheet_1.pdf]

Appendix

Supplementary Table A1: PTMPs viewed as effective

		Responses*	
**Theme**	**Subtheme**	**Mining sector**	**EMS sector**
**Effective**	Multiple counselling sessions	"Yes, long after the accident happened the guy who was with him there and saw what happened, still went for treatment and even my team leader afterwards went to HR to get help." (P8. Male. 25 years old)	"I went to five sessions with a clinical psychologist …" (28. Female. 32 years old)
	Face to face counselling	"The HR department handles those things, but they sent injured people to the doctors who then refer then to psychologists …" (P1. Male. 36 year old)	"The company providing counselling has helped me to talk about the incident. They sent me a couple of times; I feel that it is a wonderful programme and I don't have any complaints." (P16. Male. 37 years old)
	Regaining control		"You know my temper, I don't lose it that quickly anymore… it gives you control, because an angry mind is a narrow mind…" (P2. Male. 27 years old)
	Support	"… but after the social worker spoke to us we managed to understand and cope with whatever trauma we went through …" (P17. Male. 44 years old)	"We do the buddy system. Your colleague … someone who is also in EMS. It just have to be someone who you can feel … understand. Even if he does not really understand, like she says, even if he just listens." (P34. Female. 34 years old)

**No responses received from policing sector*

Supplementary Table A2: PTMPs viewed as Ineffective

		Responses*		
**Theme**	**Subtheme**	**Mining sector**	**Policing sector**	**EMS sector**
**Ineffective**	Cannot relate	"I think people know about it, but they don't trust the people to talk to them, they can't relate. It makes it pointless." (P8. Male. 25 years old)	"You don't really want to talk to them because the person that is sitting there has not been through the same situation that. He cannot empathise with you. He has never been in that situation, he has never been in a shooting, he never had to take someone's life, go out from there and now they want to tell me that they empathise with me." (P29. Male. 35 years old)	"Like the people that one talks to are not really trained to give you a better perspective." (P11. Male. 25 Years old)
	Lack of involvement	"We need someone that can talk face-to-face with us that can see our pain, not over the phone." (P8. Male 25 years old)	"And sometimes they are not even there, they don't exist. They just exist on paper or something." (P26. Male. 33 years old)	"I don't think the guys from the counselling company understand what we are doing, they sit behind a desk …" (P20. Male. 33 years old)
	Lack of supervisor support	"We need our supervisors to be close to us, not to close to us if it is something for the production only, even if the event of the death of someone, the event of bad things we need them to come close to us, all the time." (P 19. Male. 34 years old)	"So I think professionalism in terms of supporting, all the structures is poor." (P5. Male. 44 years old)	
	Unavailability of counselling	"I also feel that I require help weekly and I do not always have the capacity every week, or just when I need someone, to talk to a psychologist." (P 4. Female. 36 years old)	"This people don't interact with us, we only see them, the only time you are going to see is when something big happens and then they want to be on board, otherwise they are absent all the time." (P25. Male. 39 years)	"No, nothing, nothing at all … there is no support available, no trauma programme …" (P12. Male. 43 years old)

	Lack of specialised skills	“She sometimes does not answer her phone and by the time she phones back, I have received help elsewhere.” (P 4. Female. 36 years old)	“I think they need to be empowered. They have knowledge that is.. too much limited.” (P4. Male. 31 years old)	“I don’t think the guys from the counselling company understand what we are doing..” (P20. Male. 33 years old)
	Premature resuming of duties	“We had a fatal today, the following day our supervisors gave us the instruction on the same place we had the fatal …” P18. Male. 41 years old)		
	Single dimension approach		“Our trauma programme.. basically it is based on a principle of debriefing.. they ask you questions and look into your eyes and then you go home. Within the police service, debriefing and that’s the end of the story.” (P13. Male. 51 years old)	
